# Microarrays, Enzymatic Assays, and MALDI-MS for Determining Specific Alterations to Mitochondrial Electron Transport Chain Activity, ROS Formation, and Lipid Composition in a Monkey Model of Parkinson’s Disease

**DOI:** 10.3390/ijms24065470

**Published:** 2023-03-13

**Authors:** María Dolores García-Fernández, Ane Larrea, Roberto Fernández, Rafael Rodríguez-Puertas, Egoitz Astigarraga, Iván Manuel, Gabriel Barreda-Gómez

**Affiliations:** 1 Research and Development Department, IMG Pharma Biotech S.L., 48160 Derio, Spain; 2 Department of Pharmacology, Faculty of Medicine and Nursing, University of the Basque Country, UPV/EHU, 48940 Leioa, Spain; 3 Neurodegenerative Diseases, Biocruces, Bizkaia Health Research Institute, 48903 Barakaldo, Spain

**Keywords:** microarray, mitochondria, Parkinson’s disease, MALDI imaging mass spectrometry

## Abstract

Multiple evidences suggest that mitochondrial dysfunction is implicated in the pathogenesis of Parkinson’s disease via the selective cell death of dopaminergic neurons, such as that which occurs after prolonged exposure to the mitochondrial electron transport chain (ETC) complex I inhibitor, 1-methyl-4-phenyl-1,2,3,6-tetrahydropyrine (MPTP). However, the effects of chronic MPTP on the ETC complexes and on enzymes of lipid metabolism have not yet been thoroughly determined. To face these questions, the enzymatic activities of ETC complexes and the lipidomic profile of MPTP-treated non-human primate samples were determined using cell membrane microarrays from different brain areas and tissues. MPTP treatment induced an increase in complex II activity in the olfactory bulb, putamen, caudate, and *substantia nigra*, where a decrease in complex IV activity was observed. The lipidomic profile was also altered in these areas, with a reduction in the phosphatidylserine (38:1) content being especially relevant. Thus, MPTP treatment not only modulates ETC enzymes, but also seems to alter other mitochondrial enzymes that regulate the lipid metabolism. Moreover, these results show that a combination of cell membrane microarrays, enzymatic assays, and MALDI-MS provides a powerful tool for identifying and validating new therapeutic targets that might accelerate the drug discovery process.

## 1. Introduction

Parkinson’s disease (PD) is a chronic and progressive neurodegenerative disorder that alters the nervous system. PD is included within the movement disorders, and is the second most prevalent neurological disease [1]. There is no single cause that explains PD, and, in fact, several factors related to its etiology have been identified. It could be due to a combination of genetic, metabolic, environmental, and aging-related factors [2,3]. PD patients present a degeneration of dopaminergic neurons in the *substantia nigra* that seems to be related to the formation of α-synuclein protein aggregates [4]. It is estimated that a 70–80% loss of dopaminergic neurons is required to trigger the disease [5]. As a result, a decrease in dopamine is produced, and therefore, in the information transfer for the performance of movements. The characteristic symptoms are resting tremor, rigidity, akinesia, symptoms of unilateral predominance, fatigue, and balance disturbance [6]. PD also includes neuropsychiatric symptoms, autonomic nervous system dysfunction, pain, and diarrhea [7,8]. In addition, complex I deficits in the *substantia nigra* and the frontal cortex have been documented.

In 1970, the synthetic toxin 1-methyl-4-phenyl-1,2,3,6-tetrahydropyridine (MPTP) was found to produce PD-like motor symptoms in humans. It was subsequently determined that its active metabolite, MPP^+^, could cross the blood–brain barrier, and bind to dopaminergic neurons. MPP^+^, once bound to complex I, inhibits the mitochondrial electron chain [9], although with less potency than the specific inhibitors rotenone or dihydrorotenone. Consequently, ATP formation is reduced, and reactive oxygen species levels are increased. This discovery allowed for the use of complex I inhibitors for the development of animal models of PD [10]. Inhibitors also produce the dysregulation of several mitochondrial genes (five subunits of complex I, among others), and the degeneration of *substantia nigra* neurons, through apoptotic activation pathways [11].

The mitochondria are organelles that are present in most eukaryotic cells, although their amount increases in tissues that require more energy, such as the nervous system or muscle tissue [12]. A mitochondrion is composed of an outer membrane (EMM) and an inner membrane (IMM), with the intermembrane space (IMS) between them. The IMM contains different complexes that form the mitochondrial electron transport chain, which is involved in ATP synthesis via oxidative phosphorylation [13]. Mitochondria generate 90% of cellular energy in the form of ATP. Mitochondria also participates in processes of calcium regulation, the formation of reactive oxygen species (ROS), and cell apoptosis [14]. Altered mitochondrial functions have also been described in neurodegenerative and oncological pathologies, and in type 2 diabetes mellitus [15].

The complex I, named NADH dehydrogenase or NADH ubiquinone oxidoreductase, oxidizes to NAD+ through the transfer of two electrons to the ubiquinone molecule, which reduces to ubiquinol. This results in the transport of four protons to IMS [13]. Electrons are transported to complex III, cytochrome bc1, or the ubiquinone complex via coenzyme Q [16]. Complex III catalyzes the transfer of electrons from ubiquinol to cytochrome C. This complex also transports four protons to the IMS. Next, the two electrons are directed to complex IV, cytochrome C oxidase, via cytochrome C; where electron transfer from the reduced cytochrome C to molecular oxygen (O_2_) is catalyzed, resulting in water molecules (H_2_O), and thus, completing the oxidation–reduction process of the mitochondrial electron transport chain [17]. The protons transported by this complex, in this case, are two [18]. Complex V, ATP synthase, results in the synthesis of ATP thanks to the energy of the transmembrane electrochemical gradient caused by the passage of protons from complexes I, III, and IV [19].

The main intracellular source of ROS generation is the mitochondria, due to electron transfer during ATP production [20]. ROS are organic and inorganic small sized molecules that have an unpaired electron in the outermost orbital [21]. Therefore, ROS are highly reactive. Their formation is due to the by-product of oxygen metabolism, mainly in the mitochondrial electron transport chain, and there are three species: superoxide anion (O_2_^−^), hydrogen peroxide (H_2_O_2_), and hydroxyl (HO^−^) [22]. Their formation occurs mainly in complexes I and III, while their production in complex II is reduced due to the structure of the enzyme. Various enzymatic (superoxide dismutase, catalase, …), and non-enzymatic antioxidant systems (melatonin, vitamin D, vitamin E, …) are also involved and regulate the cellular concentration of ROS [23]. Therefore, the presence of imbalances between the production and antioxidant mechanisms can lead to oxidative stress [24]. 

Oxidative stress and the pathophysiology of PD are closely related. The autoxidation of dopamine and treatment with L-Dopa causes an increase in free radicals such as H_2_O_2_ and hydroxyl OH^−^ [25]. Under normal conditions, glutathione (GSH) peroxidase inactivates H_2_O_2_, but this antioxidant system seems to be downregulated in the brain in the early stages of the disease, leading to membrane lipid peroxidation and cell apoptosis [26,27]. This decrease over the GHS system activity may be due to oxidative stress or mitochondrial alterations [28]. In addition, the literature highlights the alterations of mitochondrial Complex I in patients with PD, the decrease of which produces the alteration of ATP synthesis, and consequently the source of cellular energy [29,30]. On the other hand, oxidative stress may also be due to nitric oxide (NO). NO can lead to the formation of peroxynitrite (ONOO^−^), which produces the interaction of NO with superoxide radicals [31,32]. NO can also inhibit the mitochondrial respiratory chain, especially in complex IV [33].

Thus, the aim of this study was to determine the density of mitochondrial complex I, superoxide formation mediated by NADH and succinate dehydrogenases, and cytochrome c oxidase activity, as well as to determine the lipid profile in a monkey model of PD, *Macaca fascicularis* treated with MPTP, using cell membrane microarrays (CMMAs). CMMAs used in this study consisted of a collection of membranes isolated from monkeys’ tissues and brain areas, which maintain the membrane environment and protein functionality, enabling their use in these assays. 

## 2. Results

### 2.1. Density of the Mitochondrial Complex I Using [^3^H]-dihydrorotenone 

**Figure 1:**
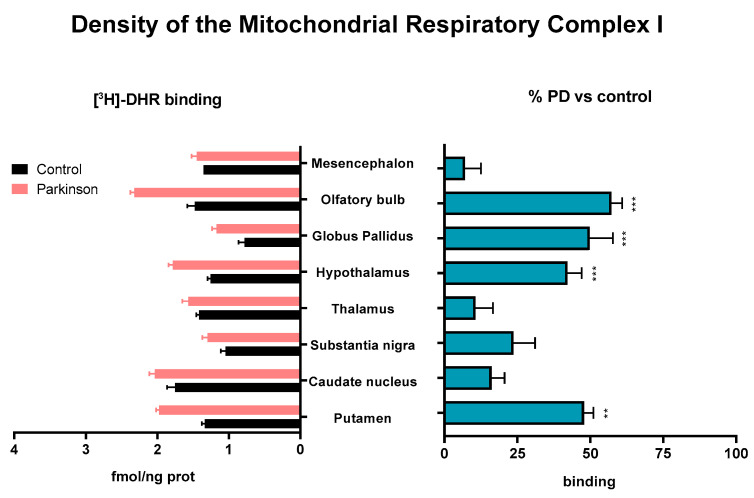
[^3^H]-DHR binding sites determined on membranes isolated from different brain areas of control and MPTP-treated animals. Results are expressed as [^3^H]-DHR density (fmol/ng prot, **left histogram**), and as percentages of change with respect to the control group (% PD vs. control, **right histogram**) in Mean ± SEM. The Shapiro–Wilk test was performed to test normality. For data with a Gaussian distribution, a one-way ANOVA statistical test with α: 0.05 was performed. For data without a normal distribution, a Kruskal–Wallis statistical test with α: 0.05 was performed. ** *p* < 0.01; *** *p* < 0.001.

The densities of binding sites of the specific inhibitor of the complex I, [^3^H]-dihydrorotenone ([^3^H]-DHR), were significantly enhanced in various nuclei of MTPT-treated monkeys. A significant increase was observed in the olfactory bulb (57 ± 4% vs. Control); in the *globus pallidus* (50 ± 8% vs. Control), in the *hypothalamus* (42 ± 5% vs. Control), and in the putamen (33 ± 15% vs. Control) (Figure 1).

### 2.2. Superoxide Formation Evoked by NADH Dehydrogenase Activity in Samples from MPTP-Treated and Control Monkeys

**Figure 2:**
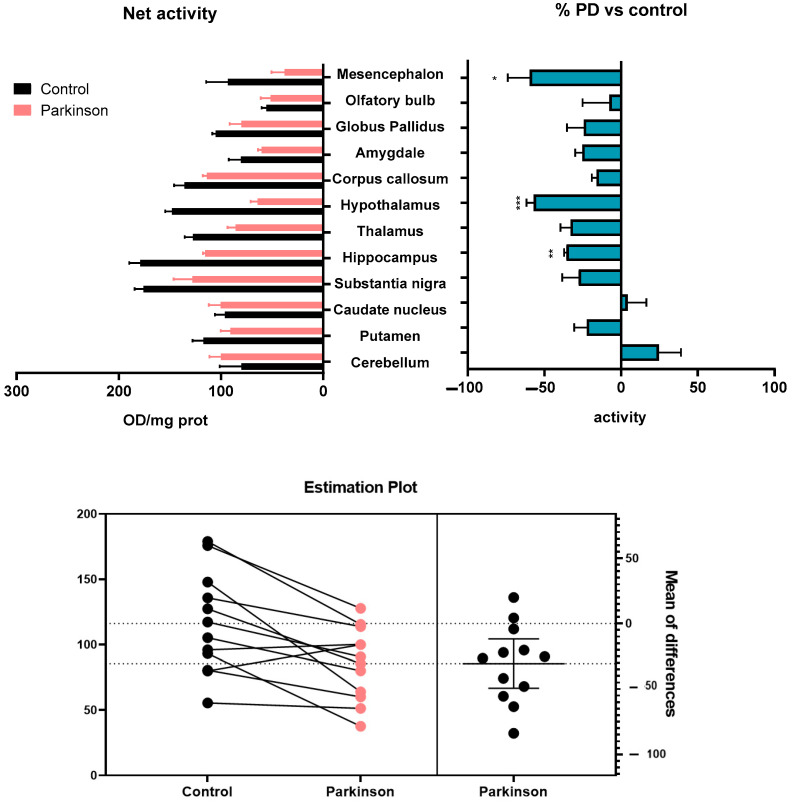
Superoxide formation evoked by NADH after the inhibition of cytochrome c oxidase activity with sodium azide in brain areas and tissues of control, and MPTP-treated animals. Results are expressed as net superoxide formation (Net activity, **top left histogram**), and as percentages of change with respect to control group (% PD vs. control, **top right histogram**) in Mean ± SEM. Shapiro–Wilk test was performed to test normality. Multiple Mann–Whitney tests with α: 0.05 were performed. * *p* < 0.05; ** *p* < 0.01; *** *p* < 0.001. The estimation plot resulting from the paired two-tailed t-test shows the magnitude of the effect after the MPTP treatment, along with a visual representation of the precision (**bottom**).

The superoxide production triggered by NADH after the inhibition of cytochrome c oxidase activity with sodium azide was significantly reduced in the *hypothalamus* of MPTP-treated monkeys, compared to control animals (*p* < 0.05). A general reduction was also observed in brain areas such as the *mesencephalon*, *hippocampus*, *thalamus*, and *substantia nigra*. The analysis of both groups using a paired two-tailed t-test found a significant reduction in superoxide production triggered by NADH (*p* = 0.0044) in MPTP-treated monkeys (Figure 2).

### 2.3. Superoxide Formation Evoked by Succinate Dehydrogenase Activity in Samples from MPTP-Treated and Control Monkeys

**Figure 3:**
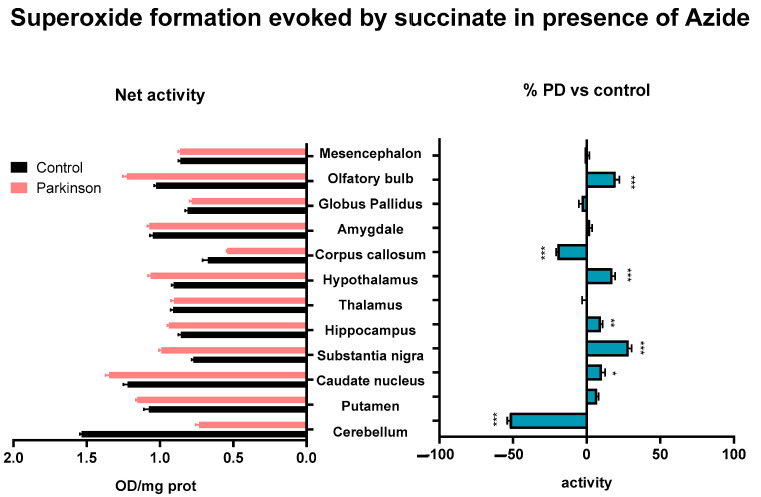
Superoxide formation evoked by succinate after the inhibition of cytochrome c oxidase activity with sodium azide in brain areas of control and MPTP-treated animals. Results are expressed as net superoxide formation (Net activity, **left histogram**), and as percentages of change with respect to control group (% PD vs. control, **right histogram**) in Mean ± SEM. Shapiro–Wilk test was performed to test normality. Multiple Mann–Whitney tests with α: 0.05 were performed. * *p* < 0.05; ** *p* < 0.01; *** *p* < 0.001.

The activity of the mitochondrial ETC complex II, succinate dehydrogenase, was reduced at the *cerebellum*, and *corpus callosum* (*p* < 0.001) in MPTP-treated monkeys, compared to the control animals. By contrast, the activity was increased at the caudate nucleus (*p* < 0.05), *hippocampus* (*p* < 0.05), *substantia nigra* (*p* < 0.001), *hypothalamus* (*p* < 0.001), and olfactory bulb (*p* < 0.001) (Figure 3).

### 2.4. Cytochrome C Oxidase Activity in Samples from MPTP-Treated and Control Monkeys

**Figure 4:**
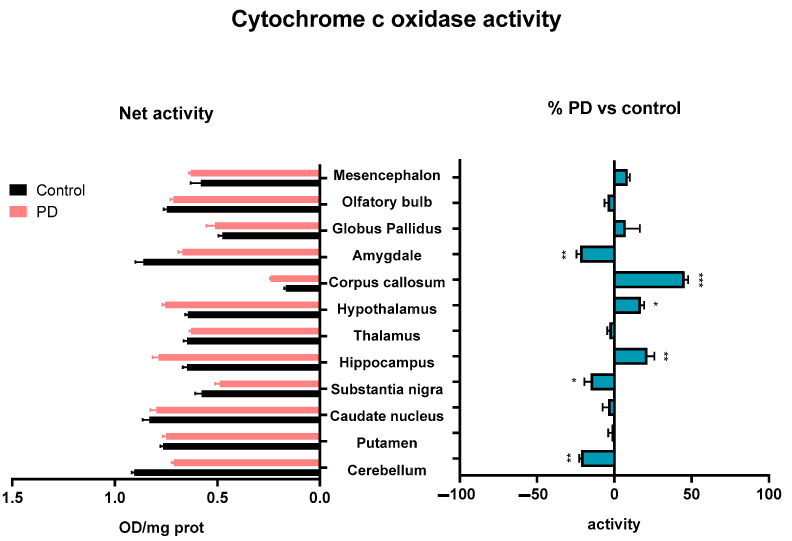
Cytochrome c oxidase activity determined on membranes isolated from different brain areas of control and MPTP-treated animals. Results are expressed as net cytochrome c oxidase activity (Net activity, **left histogram**), and as percentages of change with respect to control group (% PD vs. control, **right histogram**) in Mean ± SEM. Shapiro–Wilk test was performed to test normality. For data with a Gaussian distribution, a one-way ANOVA statistical test with α: 0.05 was performed. For data without a normal distribution, a Kruskal–Wallis statistical test with α: 0.05 was performed. * *p* < 0.05; ** *p* < 0.01; *** *p* < 0.001.

Regarding the mitochondrial activity of the ETC complex IV, cytochrome c oxidase, MPTP treatment promoted an enhancement on the *hypothalamus* (*p* < 0.05), *hippocampus* (*p* < 0.01), and *corpus callosum* (*p* < 0.001). In contrast, a decrease was observed in the *substantia nigra* (*p* < 0.05), *cerebellum* (*p* < 0.01), and amygdala (*p* < 0.01) (Figure 4).

### 2.5. Analysis of the Lipid Composition of Brain Areas and Tissues after MPTP Exposure

**Figure 5:**
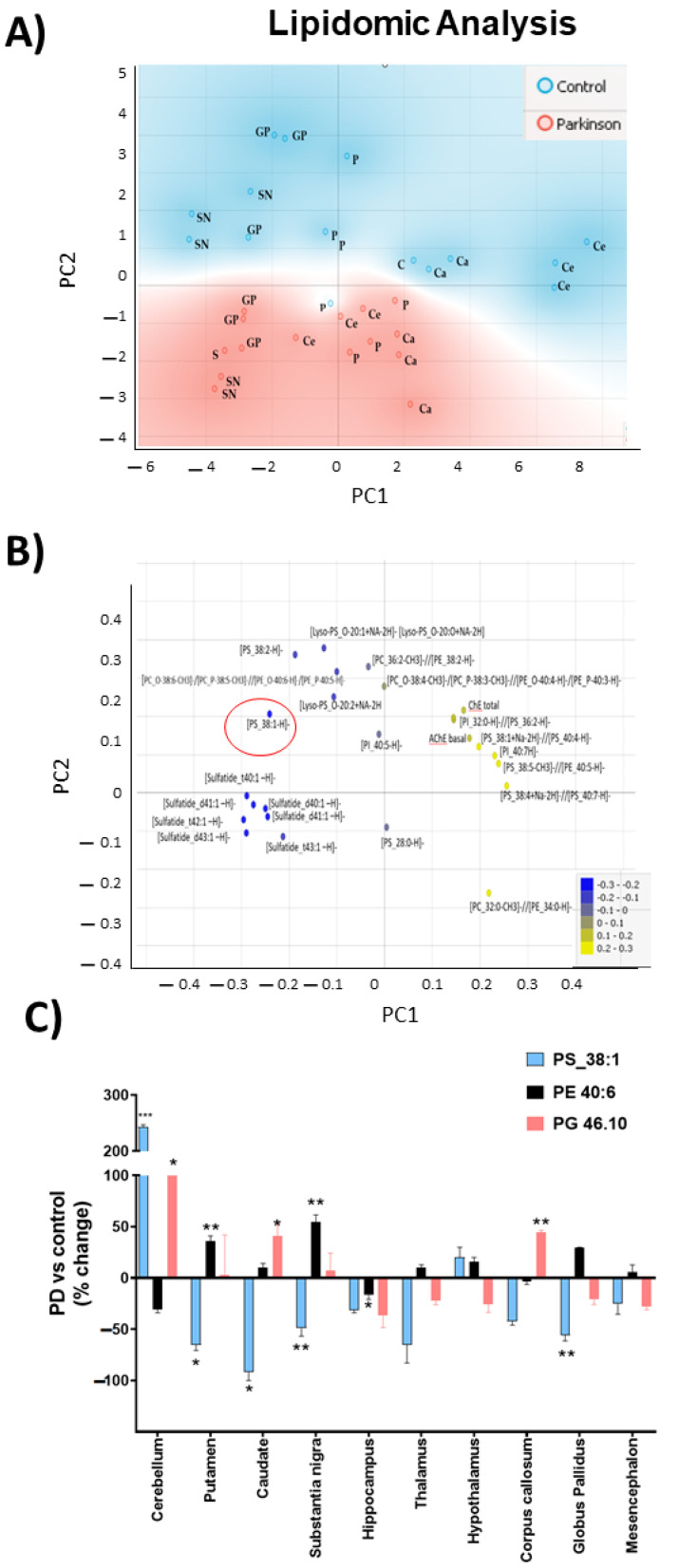
Lipidomic analysis of MPTP-treated and control monkeys. (**A**) Principal components analysis (PCA) of different brains areas (GP: *globus pallidus*, C: *cerebellum*, P: putamen, Ca: caudate, and SN: *substantia nigra*) Blue areas represent control monkeys and red areas represent MPTP-monkeys. (**B**) Loadings plot of lipidic variables. The 25 best-ranked lipids were obtained through a one-way ANOVA test. Red circle highlights [PS 38:1] (**C**) Changes in relative abundances of [PS 38:1], [PE 40:6], and [PG 46:10]. Results are expressed as percentages of change with respect to control group (mean ± SEM) (Table S2). To test normality, Barlett test was performed with α: 0.05. Parametric variables were compared using one-way ANOVA statistical test with Bonferroni post hoc, and α: 0.05 was performed. No parametric variables were compared when Kruskal–Wallis statistical test with α: 0.05 was performed: * *p* < 0.05; ** *p* < 0.01; *** *p* < 0.001.

The lipid fingerprint determined via mass spectrometry revealed specific changes in MPTP-treated monkeys compared to control animals (Table S1). Significant differences were found in brain areas related to PD (*cerebellum*, putamen, caudate, and *substantia nigra*), with [phosphatidylserine 38:1] being one of the lipids with the highest contribution in the principal component analysis (PCA). A significant reduction in [PS 38:1] content was observed in these areas, except in the *cerebellum*, where it was increased (Figure 5); (Table S2). 

### 2.6. Analysis of Lipids Displaying Significant Correlations with Any of the Activities in Control and MPTP-Treated Monkeys

To look after lipids with the same behaviors as the activities, we calculated the -fold change between the Parkinson and Control models for each lipid in each tissue. Then, we compared it against the -fold change obtained for the activities, using Pearson correlation as a distance. Highly positive values in the correlation mean that changes between the Parkinson and the control group were in the same direction in lipid and activity; meanwhile, highly negative values mean that when the lipid increases, the activity descends. To ensure that the correlation was not casual, we considered the p-value of each correlation, taking into account only those with a *p*-value of less than 0.01.

**Figure 6:**
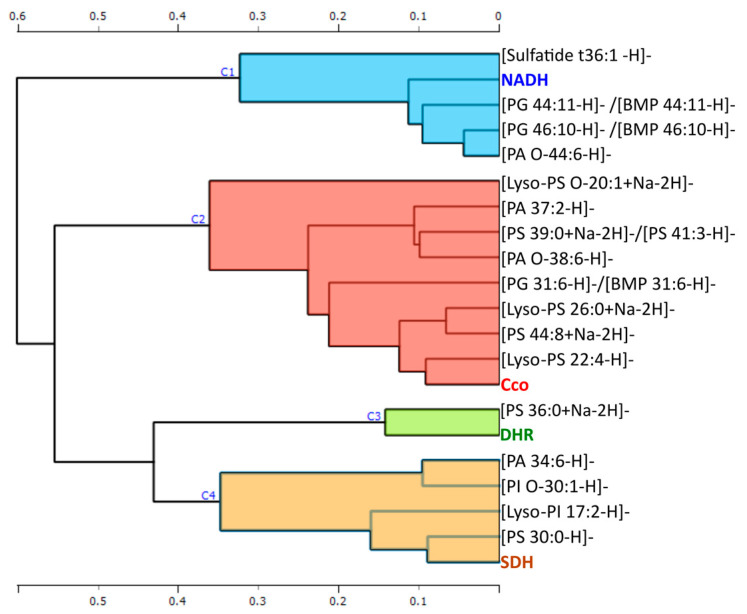

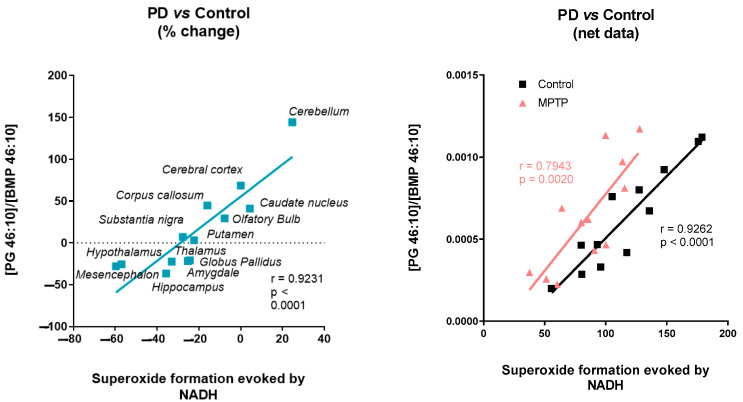
The figure shows a Pearson correlation-based hierarchical clustering of the variables that have significant correlations with any of the activities.

Once we discarded the variables that did not present significant correlations with any of the activities, we performed a Pearson correlation-based hierarchical clustering of the variables, segmenting them into four clusters. All the variables included in each cluster presented similar behaviors (Figure 6).

## 3. Discussion

In 1982, several young people were hospitalized in California in a state of paralysis, with symptoms similar to Parkinson’s disease, after the ingestion of a drug synthesized by one of them, MPTP. MPTP causes the inhibition of mitochondrial electron transport chain complex I, through its active metabolite MPP^+^. This effect is triggered by binding to the same site or to a site close to rotenone, a specific mitochondrial complex I inhibitor, that blocks NADH dehydrogenase activity [34]. Thus, it prevents the correct reduction of ubiquinone. The binding of MPP^+^ to complex I causes an interruption in electron flow, and consequently, a decrease in ATP synthesis and an increase in ROS production, and leads to the death of dopaminergic neurons [10,35]. However, the effects of chronic MPTP on ETC complexes and on enzymes of lipid metabolism have not yet been thoroughly determined. Therefore, here, we focus on the analysis of the actions of MPTP treatment on mitochondrial ETC complexes, superoxide formation, and cell membrane lipid composition.

The determined density of mitochondrial complex I, using the specific [^3^H]-DHR radioligand [36], revealed an increase in the density of complex I in the membranes of putamen, *hypothalamus*, *globus pallidus*, and the olfactory bulb of MPTP-treated monkeys. By contrast, a reduction of approximately 25–30% in mitochondrial complex I activity has been reported in the *substantia nigra* and the frontal cortex of PD brains [37,38,39]. In addition, a 33% decrease in the 8 kDa subunit of mitochondrial complex I in the frontal cortex of patients with PD has been described, an effect that could be due to the reduction in the stability of the subunits of this complex caused by oxidative stress [38]. This enhancement in highly relevant areas in PD may be due to the compensatory mechanisms of the mitochondria to palliate the inhibition triggered by the toxin. In this sense, an increase in succinate dehydrogenase activity [34,35,36] was also found in the *substantia nigra*, *hippocampus*, caudate, olfactory bulb, and *hypothalamus*, while a reduction was observed in the *corpus callosum*, and the *cerebellum*. The cytochrome c oxidase activity was also significantly reduced in the *cerebellum*, as well as in frontal cortex, *substantia nigra*, and amygdala, whilst it was increased in others such as the *hippocampus*, *hypothalamus*, and *corpus callosum*. Particularly in the *substantia nigra*, the MPTP-treated group displayed an increase in succinate dehydrogenase activity, and a decrease in cytochrome c oxidase activity. A study in lesioned rats with 6-hydroxydopamine (6-OHDA) described an enhancement of succinate dehydrogenase activity in this area, as well as an elevation in cytochrome c oxidase activity [40]. Similarly, with respect to cytochrome c oxidase activity in MPTP-treated monkeys, an increase in the *substantia nigra* has been described [41], although other authors found no changes in cytochrome c oxidase activity in this area [42]. These discrepancies may be caused by differences in the degree of the degeneration of the *substantia nigra* due to the different protocols and administration patterns used, since, as mentioned above, this region is one of the most sensitive to this toxin. In general, an increase in mitochondrial enzyme activities has been described in 6-OHDA-lesioned rats, as well as in primates treated with MPTP in the subthalamic nucleus and its projection nuclei [41], similar to that observed in membrane microarrays. However, there are no studies in the literature on the enzymatic activities in the rest of the tissues arranged in membrane microarrays, and so complementary studies will be necessary to confirm the observed changes.

Together with mitochondrial complexes, the mitochondrial dysfunction found in PD also depends on a variety of environmental factors, genes, and lipids [43,44]. Experimental evidence highlights the important role of lipids in pathological pathways that lead to this neurodegenerative disease [44]. Lipid alterations found in experimental models expressing PD-related genes, as well as those reported in idiopathic PD, suggest an important role in the molecular mechanisms of neuronal toxicity, as lipid inducers of proteinopathy, in which altered lipid homeostasis could triggers the accumulation of aberrant proteins [43]. Alterations in the autophagy-lysosomal pathway are a common feature of neurodegenerative disorders such as PD. Indeed, genetic risk factors associated with PD include mutations in certain lysosomal proteins, such as those encoding the genes LRRK2 or GBA1, that trigger alterations in lipid metabolism, leading to the vesicular membrane accumulation of certain lipids such as bis(monoacylglycero)phosphates (BMP) [44,45,46]. These lipid alterations can also be triggered by the accumulation of aberrant proteins such as α-synuclein, affecting processes such as exosome formation [46]. According to these studies, we observed specific alterations in the lipid content of Parkinsonian monkeys that clearly discriminated them from the control group in the PCA. Several sulfatides were affected by the MPTP treatment, together with sphingomyelin [SM d38:1] and phosphatidylserine [PS 28:0], among others. Moreover, phosphatidylserines with longer acyl chains such as [PS 36:2], [PS 38:1], and [PS 38:2] were also altered in this primate model of PD, together with specific lysophosphatidylserines, phosphatidylcholines, or phosphatidylinositols. One of the lipids with the highest contribution in the PCA was [PS 38:1]. This phosphatidylserine was significantly reduced in the *globus pallidus*, putamen, caudate, and *substantia nigra*; and the brain areas mainly affected in PD, in contrast to *cerebellum*, a relative well-preserved area in PD patients, where it was increased. This result contrasts with the higher PS concentration determined in the frontal cortex of PD at early stages [47,48], in skin fibroblasts from Parkinson-mutated patients [49], or in the brains of PD animal models [46,48]. Strikingly, PS supplementation also appears to be beneficial for PD patients, significantly improving some symptoms, such as motivation, anxiety, and affectivity [50]. In addition, it has been described that PS reverses memory impairment in the reserpine-induced PD rat model [51], although it does not improve cognitive impairment in the MPTP model [52]. These discrepancies may be due to differences in the degree and stage of degeneration induced by the different protocols and administration regimens used. However, the specific mechanism that PS plays in PD still remains unclear and requires further specific studies that exceed the goal of this work.

Finally, we investigate the relationship between enzymatic targets and lipid species in order to find the structural partners, allosteric modulators, or metabolic intermediates of a specific pathway related to PD. In this context, the hierarchical clustering of the variables based on Pearson correlation found a specific cluster of lipids for each of the protein targets studied: cytochrome c oxidase activity, the density of complex I, and superoxide formation evoked by succinate and NADH. These results seem to indicate that the superoxide formation triggered by NADH is not only dependent on the mitochondrial ETC, as they are being classified in different clusters. Moreover, the density of complex I determined with the specific radiolabeled inhibitor [^3^H]-dihydrorotenone increased in several areas where a reduction in NADH-evoked superoxide formation was observed, suggesting the involvement of other enzymes than mitochondrial complex I. Although mitochondrial ETC is one the main sources of superoxide [53,54,55,56], ROS can also be produced by the NADPH oxidase (NOX) enzyme family [53], dual oxidase (DUOX) [54], monoamine oxidase (MAO) [55], and peroxisomes [56], among others. ROS and reactive nitrogen species are among the main intracellular signal transducers that trigger autophagy [57]. Alterations in the autophagy-lysosomal pathway are a common feature of neurodegenerative disorders. In this sense, NADH-dependent superoxide formation was significantly correlated with a set of lipids, among which [PG 46:10]/[BMP 46:10] stands out. BMP are intermediates generated in the lysosome during lipid metabolism by certain lysosomal proteins, such as those encoding the genes LRRK2 or GBA1. Mutations in these genes trigger the accumulation of certain lysosomal lipids such as BMP [45,58]. Interestingly, the concentration of BMP in urinary exosomes correlates with the cognitive decline observed in certain PD patients [45]. 

In summary, the combination of microarray technology, enzymatic assays, and MALDI-MS enables the study of specific alterations on mitochondrial enzymes´ activities, ROS formation, and lipid composition in a monkey model of Parkinson´s disease, owing to the reduced amount of sample required and the homogeneous production of CMMAs. CMMAs have been successfully used in the characterization and analysis of membrane proteins, not only at expression, but also at a functional activity level [59,60]. As the whole membrane is printed on these CMMAs, the integrity of the proteins remains stable over long periods of time, mainly due to the preservation of the lipid environment [61,62]. Thus, the combination of these technologies can be helpful for the identification of new targets, or even of novel biomarkers in the early stages of the drug discovery process, before clinical trials are started.

## 4. Materials and Methods

### 4.1. Drugs and Reagents

Nitrotetrazolium blue chloride (NBT), beta-nicotinamide adenine dinucleotide (NADH), rotenone, sodium succinate dibasic (SDH), cytochrome c from equine heart, and 3,3-diaminobenzidine tetrahydrochloride hydrate (DAB) were purchased from Sigma-Aldrich (Saint Louis, MO, USA). [^3^H]-dihydrorotenone was purchased from Perkin Elmer (Boston, MA, USA), and the microscales of [^3^H] used by standards from American Radiolabeled Chemical (Saint Louis, MO, USA).

### 4.2. Tissue Samples

Animal samples from *Macaca fascicularis* were supplied by Applied Medical Research Center (Navarre, Spain) according to the ethical protocols. The sample consisted of six male monkeys of 4 years of age. These six animals were divided into two groups: control and treatment. The control group consisted of three intact animals, while the treatment group (three monkeys) was administered a weekly dose of 1-methyl-4-phenyl-1,2,3,6–tetrahydropyridine toxin (MPTP) intravenously (0.25 mg/kg) for 9 weeks, and 0.30 mg/kg on the last week. Animals were housed under controlled temperature (21 °C), air (16 L/h), and 12-h light/darkness until the time of sacrifice. The monkeys were sedated with a dose of ketamine and midazolam. Once anesthetized, they were perfused transcranially with saline. Finally, the brains, and organs were dissected at 4 °C. The tissues were conserved at −80 °C.

### 4.3. Cell Membrane Microarray Fabrication

Cell membrane microarrays were composed of a collection of cell membrane homogenates isolated from different *Macaca fascicularis* tissues (caudate nucleus, *corpus callosum*, amygdala, *substantia nigra*, hippocampus, *globus pallidus*, cerebellum, thalamus, olfactory bulb, putamen, hypothalamus, midbrain, liver, and heart). Samples were homogenized using a disperser (Ultra-Turrax ^®^ T10 basic, IKA, Staufen, Germany) in 20 volumes of homogenization buffer (1 mM EGTA, 3 mM MgCl_2_, and 50 mM Tris-HCl, pH 7.4) supplemented with 250 mM sucrose. The crude homogenate was subjected to a 1500 rpm centrifugation (AllegraTM X 22R centrifuge, Beckman Coulter, Brea, CA, USA) for 5 min at 4 °C, and the resultant supernatant was collected and centrifuged at 18.000 g (Microfuge^®^ 22R centrifuge, Beckman Coulter, Brea, CA, USA) for 15 min (4 °C). The pellet was washed in 20 volumes of homogenized buffer, and recentrifuged under the same conditions. The tubes were finally decanted, and the pellets were frozen at −80 °C, except for one aliquot, which was used to determine the protein concentration. The protein concentration was determined using the Bradford method [63,64], and adjusted to the final concentration. 

Membrane homogenates were resuspended in buffer and printed onto glass slides using a non-contact microarrayer (Nanoplotter NP 2.1, Gesim Bioinstruments, Radeberg, Germany), with tip J, placing three replicates of each sample (7 nL/spot) onto preactivated glass microscope slides. Membrane homogenates of each tissue were obtained from three different individuals. Printing was carried out under controlled humidity (relative humidity 60%) at a controlled temperature of 4 °C. CMMAs were stored at −20 °C until usage [65]. CMMAs were validated before usage via different methods, including Bradford staining for protein determination, and enzyme activity assays (succinate dehydrogenase and cytochrome c oxidase) [60,65,66]. 

### 4.4. Autoradiography Analysis on Mitochondrial Complex I Using [^3^H]-DHR]

NADH:ubiquinone oxidoreductase activity assays were performed using cell membrane microarrays from different *Macaca fascicularis* tissues (caudate nucleus, *corpus callosum*, amygdala, *substantia nigra*, *hippocampus*, *globus pallidus*, *cerebellum, thalamus*, olfactory bulb, putamen, hypothalamus, and midbrain). CMMAs were dried for 15 min, and then preincubated for 30 min in the presence of buffer Tris-HCl (25 mM; pH 7.6). Later, the microarrays were incubated during 120 min with reaction solution containing NADH (200 µM), [^3^H]-DHR (5 nM) in Tris-HCl buffer (50 mM; pH 7.6; BSA 1%). Non-specific union was determined using same reaction solution in the presence of rotenone (100 µM). After the incubation time, the reaction was stopped by dipping twice in Tris-HCl (25 mM; pH 7.4) at 4 °C for 5 min. Finally, CMMAs were dried with a cold flow air (4 °C), and exposed to a radiosensitive film for 3 months in the presence of [^3^H]-microscales. To normalize the data, microarrays were exposed to previously calibrated commercial standards. The impressions of the radiosensitive films generated by the [^3^H] standards were quantified using a grating of the same characteristics. The standards allowed a calibration curve to be obtain in each experiment, where the nCi/g were calculated from the gray densities determined in each microarray. Finally, the values expressed in nCi/g were converted into molar units per tissue unit [67].

### 4.5. Determination of a Tissue-Specific Effect on Superoxide Formation Induced by Succinate Dehydrogenase Using the CMMAs of MPTP-Treated and Control Monkeys

Succinate dehydrogenase activity assays were performed using cell membrane microarrays from different *Macaca fascicularis* tissues (caudate nucleus, *corpus callosum*, amygdala, *substantia nigra*, *hippocampus*, *globus pallidus*, *cerebellum, thalamus*, olfactory bulb, putamen, *hypothalamus,* and midbrain). CMMAs were incubated in the presence of 1 mM succinate, and 1.2 mM NBT in phosphate buffer (0.05 M; pH 7.4) for 16 h at 25 °C. The reaction was started by the addition of the reagents to the CMMAs. After the incubation time, the reaction was stopped by dipping in phosphate buffer for 10 min, and next, in dH_2_O. Once dried, the CMMA color signal was acquired with an Epson V750 pro scanner (version 4.6.5.0, Seiko Epson Company, Nagano, Japan) and digital images were analyzed with the software ScanAlyze (version 2.35, Michael Eisen, Standford, CA, USA) and quantified using the software Microsoft Office Excel (version 17.0, Microsoft, Albuquerque, NM, USA).

### 4.6. Determination of Tissue-Specific Effects on Superoxide Formation Induced by Cytochrome C Oxidase Activity Using CMMAs of MPTP-Treated and Control Monkeys

Cytochrome c oxidase activity assays were performed on CMMAs from different *Macaca fascicularis* tissues (caudate nucleus, *corpus callosum*, amygdala, *substantia nigra*, *hippocampus*, *globus pallidus*, *cerebellum*, *thalamus*, olfactory bulb, putamen, *hypothalamus*, and midbrain). CMMAs were incubated in the presence of 1.3 mM DAB in phosphate buffer (0.1 M; pH 7.4) for 16 h at 25 °C. The reaction was started with the addition of the reagents to the CMMAs. After the incubation time, the reaction was stopped by dipping in phosphate buffer for 10 min, and next, in dH_2_O. Once dried, the CMMA color signal was acquired with an Epson V750 pro scanner (version 4.6.5.0, Seiko Epson Company, Nagano, Japan), and digital images were analyzed using the software ScanAlyze (version 2.35, Michael Eisen, Standford, CA, USA), and quantified using the software Microsoft Office Excel (version 17.0, Microsoft, Albuquerque, NM, USA).

### 4.7. MALDI-MS Lipidomic Analysis

The Cell Membrane Microarrays were covered with a suitable matrix with the aid of a standard glass sublimator (Ace Glass 8233, Vineland, NJ, USA), producing a uniform film of approximately 0.2 mg/cm^2^. For positive-ion, and negative-ion modes, 2-mercaptobenzothiazole (MBT) and 1,5-diaminoaphtalene (DAN) were used, respectively [61,68]. The CMMAs were scanned, as in the MALDI imaging experiment. The area of the array was explored following a grid coordinate separated by 250 µm; as each spot has a diameter of 280 µm, six pixels were recorded at each spot. The mass spectrometer used was an LTQ-Orbitrap XL (Thermo Fisher Scientific, Waltham, MA, USA), equipped with a MALDI source with N2 laser (60 Hz, 100 µJ/pulse maximum power output). The laser spot is an ellipsoid of approximately 50–60 µm × 140–160 µm. Two microscans of 10 shots/pixel were used, with a laser power output of 20 µJ for MS+, 30 µJ for MS-, and a resolution of 40.0000 full width at half maximum (FWHM). Data loading included spectra normalization via total ion current (TIC), spectra alignment, and peak picking, filtering all of the *m*/*z* with an intensity < 0.5% of the strongest peak in the spectrum. 

The MALDI spectra were aligned using MATLAB (MathWorks, Natick, MA, USA). Lipid assignment was performed using a homemade database and the Lipid Maps LMSD database. For the MALDI data analysis, the MS+ and MS−data were normalized separately, and then analyzed together. The matrix peaks, and isotopic distribution were removed, and the remaining peaks were normalized against their total ion current (TIC). The MS+ and MS− data were standardized using the *z*-score method, and using the following formula:

$$Z=\frac{x-\mu }{\sigma }$$

*x*: Observed value; *µ*: mean of sample; *σ*: standard deviation of the sample.

### 4.8. Data Handling and Statistical Analysis

Data handling and analysis were carried out using Microsoft Excel (version 17.0, Microsoft, New Mexico, USA) and GraphPad software (version 5, GraphPad, La Jolla, CA, EE.UU). 

For microarrays, the analysis data obtained were normalized to the total protein amount, and expressed as the means of independent data points ± S.E.M. The normality of the data was tested using the Shapiro–Wilk statistical test, with α set at 0.05. For Gaussian distributed data, a statistical analysis one-way ANOVA test, two-tailed with Bonferroni post hoc, was performed, and α was set at 0.05. Statistical differences were indicated by *p*-values ≤ 0.05.

## 5. Conclusions

MPTP-treated monkeys displayed a high density of mitochondrial complex I, contrasting with the reduced capacity of superoxide production triggered by NADH dehydrogenase activities. In addition, succinate dehydrogenase and cytochrome c oxidase activities were also altered in this animal model of PD, along with certain lipid species. Moreover, significant correlations were identified between membrane lipids and enzyme activities, some of which were also altered in MPTP-treated monkeys.

CMMAs are useful for determining the expression and activity of membrane proteins through enzymatic activities. 

CMMAs can reduce intra-experimental variability, the numbers of samples and reagents, and the durations of experiments and analyses.

MPTP treatment modulated the mitochondrial electron transport chain enzymes, and seemed to alter other mitochondrial enzymes that regulate lipid metabolism. 

## Data Availability

The data supporting the findings of this study are available from the corresponding author, Gabriel Barreda-Gómez, upon reasonable request.

## Supplementary Materials

The following supporting information can be downloaded at: https://www.mdpi.com/article/10.3390/ijms24065470/s1.
