# Supplementary material for: Microarrays, Enzymatic Assays, and MALDI-MS for Determining Specific Alterations to Mitochondrial Electron Transport Chain Activity, ROS Formation, and Lipid Composition in a Monkey Model of Parkinson’s Disease
Source: Int J Mol Sci. 2023 Mar 13;24(6):5470. doi: 10.3390/ijms24065470 (PMC10049643; doi:10.3390/ijms24065470)
Supplement: Supplementary file 1 [file ijms-24-05470-s001.zip › ijms-2167407-supplementary.pdf]

International Journal of *Molecular Sciences*

MDPI

Table S1. 50 most significant lipid species in the paired t-test between control and Parkinson's models. The first column (m/z) shows the experimentally detected mass of the assigned lipid species; m/z (teor) is the calculated mass of the lipid species; error (Da) is the error. in Da. between the experimental and calculated mass of the lipid species; error (ppm) shows the error in parts per million; Sig is the significance of the comparison in the test (*p < 0. 05;**p < 0. 01 and***p < 0. 01: ***).p is the p-value in the test(*p < 0. 05;**p < 0. 01 and***p < 0. 01: ***), and FC (MPD-MC)/MC is the Fold change between the Parkinson (MPD) and Control (MC) models

Asigned Lipid Specie	m/z	m/z (teor)	Error (Da)	Error (ppm)	Sig	P	FC (MDP-MC)/(MC)
[PS 37:2-H]-	800.5456	800.5448	−0.0008	−0.99	***	0.001	−54.0%
[PS 38:1−H]−	816.5772	816.5761	−0.0011	−1.31	**	0.002	−32.8%
[PS 40:1+Na−2H]−/[PS 42:4−H]−	866.5927	866.5894	−0.0033	−3.81	**	0.003	−22.4%
[PG 43:2−H]−	871.6445	871.6434	−0.0011	−1.23	**	0.003	263.6%
[PG 44:12−H]−	865.5007	865.5025	0.0019	2.15	**	0.004	−29.2%
[PS 38:2−H]−	814.5613	814.5604	−0.0009	−1.09	**	0.005	−28.3%
[PS 36:0+Na−2H]−	812.5455	812.5424	−0.0031	−3.77	*	0.011	−25.3%
[PS 40:2−H]−	842.5916	842.5917	0.0001	0.13	*	0.011	−34.4%
[PS 47:3+K−2H]−	976.6395	976.6415	0.0020	2.05	*	0.012	−58.6%
[PA 32:0−H]−	647.4655	647.4657	0.0003	0.39	*	0.013	41.9%
[Lyso−PS O−20:0+Na−2H]−	560.3362	560.3335	−0.0027	−4.87	*	0.015	−44.3%
[PA O−40:7−H]−/[PA P−40:6−H]−	731.5027	731.5021	−0.0006	−0.84	*	0.015	265.4%
[PS 37:1−H]−	802.5614	802.5604	−0.0010	−1.21	*	0.015	−26.0%
[Sulfatide t43:1 −H]−	920.6496	920.6501	0.0005	0.55	*	0.015	13.4%
[PE O−42:6−H]−/[PE P−42:5−H]−	804.5919	804.5913	−0.0006	−0.73	*	0.018	−20.8%
[Sulfatide t38:1 −H]−	850.5721	850.5719	−0.0001	−0.16	*	0.019	−16.5%
[PE 32:1−H]−	688.4951	688.4923	−0.0028	−4.07	*	0.020	102.5%
[PS 28:1+Na−2H]−/[PS 30:4−H]−	698.4048	698.4016	−0.0033	−4.65	*	0.020	−79.9%
[PE O−42:5−H]−/[PE P−42:4−H]−	806.6077	806.6069	−0.0008	−0.96	*	0.020	−15.1%
[PG O−34:2−H]−/[PG P−34:1−H]−	731.5222	731.5233	0.0010	1.41	*	0.022	150.3%

[PG O−34:3−H]−/[PG P−34:2−H]−	729.5076	729.5076	0.0000	−0.01	*	0.023	155.8%
[PS 38:2+Na−2H]−/[PS 40:5−H]−	836.5441	836.5424	−0.0017	−2.02	*	0.023	−29.8%
[PA 34:1−H]−	673.4812	673.4814	0.0002	0.33	*	0.023	19.8%
[PS 36:1−H]−	788.5444	788.5448	0.0004	0.46	*	0.023	−10.4%
[PE O−40:3−H]−/[PE P−40:2−H]−	782.6078	782.6069	−0.0009	−1.18	*	0.023	−42.0%
[Sulfatide t33:2 −H]−	778.4775	778.4780	0.0005	0.68	*	0.026	−53.7%
[PG O−40:4−H]−/[PG P−40:3−H]−	811.5863	811.5859	−0.0005	−0.57	*	0.032	188.0%
[PS O−38:7+K−2H]−/[PS P−38:6+K−2H]−	828.4576	828.4588	0.0012	1.42	*	0.032	−63.3%
[PA 40:3−H]−	753.5428	753.5440	0.0012	1.55	*	0.033	−60.6%
[PE 32:0−H]−	690.5085	690.5079	−0.0005	−0.78	*	0.038	72.3%
[CerP d42:1 −H]−	728.5960	728.5963	0.0003	0.45	*	0.041	266.9%
[PG O−40:3−H]−/[PG P−40:2−H]−	813.6009	813.6015	0.0006	0.71	*	0.043	57.6%
[Sulfatide d43:1 −H]−	904.6534	904.6552	0.0019	2.05	*	0.043	14.2%
[PS 29:2−H]−	688.4195	688.4196	0.0001	0.09	*	0.046	−75.8%
[PI O−30:0−H]−	767.5049	767.5081	0.0031	4.07	*	0.047	24.2%
[PE O−40:6−H]−/[PE P−40:5−H]−	776.5611	776.5600	−0.0011	−1.41	*	0.049	−11.7%
[PE 44:7−H]−	844.5867	844.5862	−0.0006	−0.65	ns	0.055	−57.1%
[PA 44:10−H]−	795.4987	795.4970	−0.0017	−2.07	ns	0.056	−83.8%
[PS 47:2+K−2H]−	978.6554	978.6572	0.0018	1.86	ns	0.056	−25.1%
[PS 48:2+K−2H]−	992.6723	992.6728	0.0005	0.50	ns	0.058	−34.4%
[PE 40:2−H]−	798.6029	798.6018	−0.0010	−1.29	ns	0.063	−36.2%
[PE 42:5−H]−	820.5869	820.5862	−0.0007	−0.85	ns	0.071	−18.7%
[PA 44:6−H]−	803.5644	803.5596	−0.0048	−5.99	ns	0.071	104.6%
[PE O−40:2−H]−/[PE P−40:1−H]−	784.6225	784.6226	0.0001	0.15	ns	0.072	−51.8%
[PI O−38:4−H]−	871.5713	871.5707	−0.0007	−0.75	ns	0.073	−38.2%
[PS O−31:1+Na−2H]−/[PS P−31:0+Na−2H]−/[PS O−33:4−H]−	726.4717	726.4693	−0.0024	−3.34	ns	0.075	−43.8%
[Sulfatide t41:1 −H]−	892.6186	892.6188	0.0002	0.24	ns	0.075	12.1%

[PS O−29:2−H]−	674.4440	674.4403	−0.0037	−5.43	ns	0.075	37.4%
[PS 34:2+Na−2H]−/[PS 36:5−H]−	780.4823	780.4798	−0.0025	−3.19	ns	0.078	−21.1%

Table S2. Changes in relative abundance of [PS 38:1], [PE 40:6], and [PG 46:10]. Results are expressed as percentages of change respect control group (mean ± SEM).

**Tissues**	**Lipids**					
	**PS 38:1**		**PE 40:6**		**PG 46:10**	
	MEAN	SEM	MEAN	SEM	MEAN	SEM
**Caudate nucleus**	8.00	8.00	9.27	4.18	40.91	12.04
**Cerebellum**	342.66	4.41	–30.86	3.00	144.15	28.24
**Corpus Callosum**	57.66	3.71	–3.48	2.98	44.58	1.90
**Globus Pallidus**	44.33	5.78	29.27	0.64	–20.71	5.23
**Hypothalamus**	120.33	9.60	15.71	4.19	–25.52	8.05
**Hippocampus**	68.66	2.60	–16.72	4.17	–36.47	11.78
**Mesencephalon**	74.66	10.17	5.75	6.88	–27.73	3.21
**Putamen**	34.66	5.36	36.19	4.78	3.09	38.76
**Substantia nigra**	51.00	7.63	54.61	6.81	7.08	17.08
**Thalamus**	35.00	18.02	10.20	2.61	–22.23	3.78
